# Temperament structures and the effectiveness of individual play in football

**DOI:** 10.3389/fpsyg.2024.1376466

**Published:** 2024-07-30

**Authors:** Łukasz Bojkowski, Maciej Tomczak

**Affiliations:** Department of Psychology, Poznan University of Physical Education, Poznań, Poland

**Keywords:** offensive effectiveness, defensive effectiveness, comprehensive effectiveness, temperament, temperament structures

## Abstract

**Background:**

Regulatory Theory of Temperament distinguishes two traits involving the time course of responses and four relating to how energy is distributed and stored. This theory enables the presentation of entire structures of temperament traits and it is relevant for assessing an individual’s ability to process stimulation and influence their performance during competitions. In our cross-sectional study, these structures are examined in relation to the offensive, defensive, and comprehensive effectiveness of an individual player’s actions.

**Methods:**

The study included 91 football players aged between 20 and 31 years, who had approximately 10 years of training experience. The Formal Characteristics of Behavior – Temperament Inventory was utilized to assess temperamental traits, while a simulation game was employed to evaluate the effectiveness of each individual player’s actions.

**Results:**

Research demonstrated that football players possessing a temperament structure that is closely aligned harmonized structure with a high capacity to process stimulation showed higher levels of individual efficacy in attack, defence, and comprehensive effectiveness of performance compared to football players with a harmonized temperament structure but a low capacity to process stimulation.

**Conclusion:**

The obtained result emphasises the significance of specific temperament structures in terms of matching the individual’s stimulation levels and the their ability to process it.

## 1 Introduction

A substantial number of contemporary research papers focus on the significance of psychology within the realm of training, including efforts to diagnose the levels of specific psychological traits and their impact on athletes’ performance effectiveness. Among these traits are temperament traits (Ghorbanzadeh et al., 2001; Bojkowski and Siejka, 2020), which are defined as inherent and relatively stable biological characteristics of the organism (Oniszczenko et al., 2003). Although temperament traits display some variation during ontogeny due to prolonged exposure to environment factors or maturation (Strelau and Zawadzki, 2014; Cyniak-Cieciura et al., 2018), they are considered to be primarily innate and relatively fixed (Strelau and Zawadzki, 2014).

Amongst the various theories of temperament, Strelau’s (2013) Regulatory Theory of Temperament (RTT) is significant. Its application in sport is beneficial due to the ability to present holistic temperament structures, allowing for a comprehensive understanding of the arrangement of traits in relation to each other. This understanding can greatly contribute to the effective functioning of individuals in sporting activities. According to the described theory, the first component of temperament comprises traits that characterize the course of reactions over time (Cyniak-Cieciura et al., 2018). The second component focuses on the energy level of behavior, which highlights the subject’s distinctive way of distributing and storing energy (Strelau and Zawadzki, 2014; Cyniak-Cieciura et al., 2018). As a result, the derived temperament structures consist of six traits (Strelau and Zawadzki, 2014; Cyniak-Cieciura et al., 2018) (Table 1).

**Table 1:** Characteristics of temperamental traits – Regulatory Theory of Temperament (RTT) (Strelau and Zawadzki, 2014; Cyniak-Cieciura et al., 2018).

Temperamental traits	Discription
Emotional reactivity (ER)	The propensity to react intensively to emotion-generated stimuli, expressed in high emotional sensitivity and low emotional endurance.
Sensory sensitivity (SS)	The capacity to perceive and react to sensory stimuli with low stimulative value.
Perseverance (PE)	The tendency to continue and to repeat behavior after the termination of the stimuli or situation evoking this behavior.
Briskness (BR)	The disposition to react quickly, to maintain a high tempo of performing activities and to shift easily in response to changes in the environment from one behavior or reaction to another.
Activity (AC)	The propensity to undertake behaviors providing strong external stimuli.
Endurance (EN)	The ability to react adequately in situations requiring long-lasting or highly stimulating activity and to work effectively under conditions of intense external stimulation.

In research examining temperamental characteristics in relation to sports activity, it has been pointed out that having appropriate levels of individual temperamental traits is one of the factors that characterises a high-performance athlete, what showed in their research: Bołdak and Guszkowska (2013) and Bojkowski and Siejka (2020). These both analyses revealed that during the selection process and the enhancement of mental skills in football, it is worth paying attention to the levels of such temperamental traits such as emotional reactivity (low), perseverance (low) and briskness (high), and activity (high). Leźnicka et al. (2017) found highly similar results on a sample of martial arts athletes. Another research shown that the specific levels of these selected temperamental traits among participants in football activities—related to competition and group cooperation (Trecroci et al., 2018)—may be linked to the effectiveness of attentional processes (Fajkowska et al., 2012), mood (Jankowski and Zajenkowski, 2012), and feelings of stress and coping styles (Wirtz et al., 2007). Moreover, the existing temperamental differences between athletes and non-athletes were pointed out by Siwek et al. (2015), while the study by Kaveshnikova et al. (2023) showed that track and field athletes show differences in temperamental traits depending on their sports specialization.

Despite the analysis of relationships between selected temperament traits and various psychological constructs (described in the preceding paragraph), researchers rarely delve into the identification of complete temperament structures. This approach, which examines functional interrelationships, captures the contribution of adopting a holistic view of a person’s temperament rather than focusing on independent traits. With regards to RTT, understanding such a structure allows for a more complete assessment of the effectiveness of behavioral regulation in terms of adequate stimulus processing in response to external stimulation. This, in turn, is believed to activate positive emotions and be associated with effective action (Strelau and Zawadzki, 2014).

In accordance with the principles of RTT, specific temperamental profiles consist of sets of traits that act as regulators of behavior in the subject-environment relationship. These profiles possess an adaptive nature and manifest most prominently under extreme and difficult conditions (Strelau, 2013; Cyniak-Cieciura et al., 2018). In the context of sports practice, this becomes particularly significant as long-term training processes involve situations of overload, conflict, or subjectively perceived obstacles on the athlete’s path to achieving their goals. Moreover, temperamental differences, both in terms of individual traits and their entire structures, can be readily observed even in children (Cyniak-Cieciura et al., 2018), thereby rendering the study results applicable across an indivdual’s lifelong engagement in activities. Therefore, to identify more and less effective temperament structures, we can utilize the typology developed by Strelau and Zawadzki (2014), who distinguished:

1 Harmonized structure with a high capacity to process stimulation – enabling effective regulation of stimulation and a stimulation-seeking orientation. This structure is characterized by elevated levels of briskness, endurance, and activity, along with reduced levels of perseverance and emotional reactivity (Strelau and Zawadzki, 2014).

Athletes characterized by a certain temperamental structure will cope well in stressful situations, so their participation in a sporting game with multiple stimuli should not negatively affect their performance. Such individuals function more easily in sports teams and are more likely to undertake new training methods. They learn more easily in the initial stages of movement mastery.

2 Harmonized structure with a low capacity to process stimulation – characterized by lower levels of briskness, endurance, and activity, as well as higher levels of perseverance and emotional reactivity. Individuals with this structure tend to perform optimally in conditions characterized by low levels of stimulation (Strelau and Zawadzki, 2014).

Sportsmen characterized by a particular temperamental structure are less resilient to changes in conditions (for example, situational changes in game or social systems) and are less resistant to prolonged stimulation. They pay more attention to emotions, may dwell on setbacks for longer and need more time to plan future actions and implement them effectively.

3 Non-harmonized structure with a high capacity to process stimulation – characterized by lower levels of briskness, emotional reactivity, and activity, and higher levels of endurance. Individuals with this structure face challenges in effectively regulating the stimulation they experience and exhibit limited discharge behaviors (Strelau and Zawadzki, 2014).

Such individuals may be considered withdrawn in interpersonal relationships and thus may have difficulty functioning in a task group (a sports team with common goals). They may find it difficult to adapt to change and take risks with a high degree of uncertainty, learning more slowly in the initial phase of sports learning, but being persistent in the improvement phase.

4 Non-harmonized structure with a low capacity to process stimulation – consisting of increased perseverance, emotional reactivity and activity, and decreased endurance. Individuals with this structure struggle to regulate stimulation effectively and are oriented toward discharging high levels of arousal. As a result, they may react with anger in certain situations (Strelau and Zawadzki, 2014).

Individuals characterized by a certain temperament structure are active in sporting activities, more willing to take on leadership activities. They can often impose a high pace of activity while being impatient and showing difficulty in adapting to the rules of teams. They may have difficulty refraining from occurring provocations in sport.

In this study, we hypothesize that people characterized by a lower level of emotional reactivity and a higher level of briskness, activity and endurance (levels of features characteristic of a harmonized temperamental structure with a high ability to process stimulation) will be more effective (offensive, defensive, and comprehensive) in individual duels in football. Therefore, our study of the temperamental structure of football players aims to explore the significance of specific temperamental trait configurations (structures) in and individual’s adaptation to the unique demands of competition, including offensive, defensive and comprehensive effectiveness during individual football competition. Additionally, we aim to understand how these traits contribute to the individual’s ability to match incoming stimulation (whether external or resulting from self-activity), their capacity to process and regulate it effectively or ineffectively.

## 2 Materials and methods

### 2.1 Participants

The survey was conducted among players representing Polish football clubs participating in league competitions at five successive levels organized by the Wielkopolski Związek Piłki Nożnej (Wielkopolska Football Association) (Table 2).

**Table 2:** Characteristics of football players in the study.

Sports level (competition level)	*N*	Age (in years)				Training experience (in years)	
		min. – max.	M	Me	SD	Me	SD
III league	28	20–28	22.8	22	1.93	11	2.65
IV league, North division	35	20–27	22.5	22	1.7	9.8	2.55
Regional league, East division	11	20–31	23.5	22	3.7	10.9	3.56
A-class, III division	11	21–30	24.3	24	2.97	10.8	3.76
B-class, II division	6	20–23	21.7	22	2.3	9.2	3.76

N – group size; min. – minimum; max. – maximum; M – medium; Me – median; SD – standard deviation.

The total research group consisted of 91 male football players aged 20 to 31 (M = 22.9, Me = 22, SD = 2.3), with an average training experience of 10.4 years (SD = 2.91). All subjects were assigned to 1 of the 13 test subgroups (7 players in each). Each subgroup consisted of players from all the above-mentioned playing levels. The research subgroups were characterized by heterogeneity, as players representing different league levels competed within each of the 13 identified subgroups. However, within each subgroup, players were matched based on similar sporting levels, ensuring that the best players were matched with the best from other subgroups, etc.

The players were tested on training pitches with artificial turf, on non-training days in very similar weather conditions (spring). In our research, the criterion for including and excluding players from the study was: representing their club in national competitions, having a minimum of 5 years’ training experience, and participation in training and sports competition in the senior age category.

The study was approved by the Research Ethics Committee of the Karol Marcinkowski University of Medical Science in Poznań (approval number 780/14; date: 02.10.2014). Our research is part of a larger project on motor and psychosocial determinants involving individual performance in football players (Bojkowski et al., 2022).

### 2.2 Measurement tools

Our study on investigating the relationship between temperamental profiles and individual effectiveness of football procedure, we used:

1 The Formal Characteristics of Behavior – Temperament Inventory (FCZ-KT) (Strelau and Zawadzki, 2014) was employed for the assessment of temperamental traits. A questionnaire by Strelau and Zawadzki (2014), constructed according to the principles of the construction of estimation scales (Brzezińska and Brzeziński, 2006), was utilized. The questionnaire comprises 120 statements on formal behavioral characteristics, organized into six groups corresponding to temperamental characteristics: emotional reactivity, sensory sensitivity, perseverance, endurance, briskness, and activity. The theoretical basis of the questionnaire lies in the RTT (Strelau and Zawadzki, 2014).
   The different measures of accuracy of the individual FCZ-KT scales are satisfactory and support the theoretical expectations regarding temperament estimation (Strelau and Zawadzki, 2014). The reliability of measurement using the FCZ-KT questionnaire, as estimated by the Cronbach’s alpha coefficient, is satisfactory for all scales: emotional reactivity (0.82–0.87), sensory sensitivity (0.70–078), briskness (0.73–0.80), perseverance (0.78–0.81), endurance (0.86–0.88), activity (0.79–0.83) (Strelau and Zawadzki, 2014).
2 A test simulation game was utilized to evaluate the player’s offensive, defensive and, comprehensive effectiveness (a one-on-one test game, using two goals, and without goalkeepers) (Ljach and Witkowski, 2004) – the playing field consisted of a square measuring 20 × 20 m, divided into two halves. Net goals were placed on the goal lines. The players in each sub-group played a “round robin” tournament (each examined subgroup conisted of seven people, thus each player played six matches). One match lasted 2 min followed by a 5–8 min break for passive or active rest (Ljach and Witkowski, 2004). An assistant referee stopped the playing time when the ball went out of play (Ljach and Witkowski, 2004). When assessing individual attacking effectiveness, an indicator of comprehensive effectiveness was used, in this case, a numerically higher result (more goals scored by a player during a one-on-one game) was treated as a better result (determining greater attack effectiveness). By contrast, for the assessment of individual defensive effectiveness, the numerically lower result (lower number of goals conceded during a one-on-one game) was considered the better one (greater defence effectiveness).
   This testing method offers a standardized way of quantifying the player’s actions during a sports game (Ljach and Witkowski, 2004). The reliability indicators developed to assess the effectiveness of one-on-one play in football are moderate to high, with a value of 0.67 for the number of goals conceded (defence), 0.86 for the number of goals scored (attack), and 0.89 for goal difference (all results were statistically significant at the level of *p* < 0.05) (Witkowski and Duda, 2005).

### 2.3 Statistical analysis

In order to analyze the temperamental structure of football players, groups with different temperamental trait profiles were identified. Cluster analysis using the k-means method (for cases) was applied, with all variables expressed on an equal stanine scale (M = 5.0; SD = 2.0). A division into two and three groups was tested, considering the interpretability of the results and the group size. The division into two groups was relatively close to the pattern of temperament trait profiles indicating a harmonized temperament structure with a high capacity to process stimulation and a harmonized temperament structure with a low capacity to process stimulation, per the Regulatory Theory of Temperament. Thus, it was ultimately considered theoretically sound. To compare the players from the separate groups (clusters) in terms of temperament traits and football performance, the t-test for independent data was applied. If the homogeneity of variance condition assessed with the Levene’s test result was not met, a t-test with correction for unequal variances (for the trait of perseverance) was applied. Furthermore, the reliability of the obtained comparisons was assessed using the U-Mann Whitney test. Test results in terms of reaching significance at the 0.05 level (i.e., significant/non-significant) were the same as the results of the t-test performed earlier. In addition, a 95% confidence interval was estimated for the difference between groups using the bootstrap percentile method with 10.000 samples. If the confidence interval does not contain a zero value then the difference between groups is statistically significant at the 0.05 level.

## 3 Results

**Figure 1:**
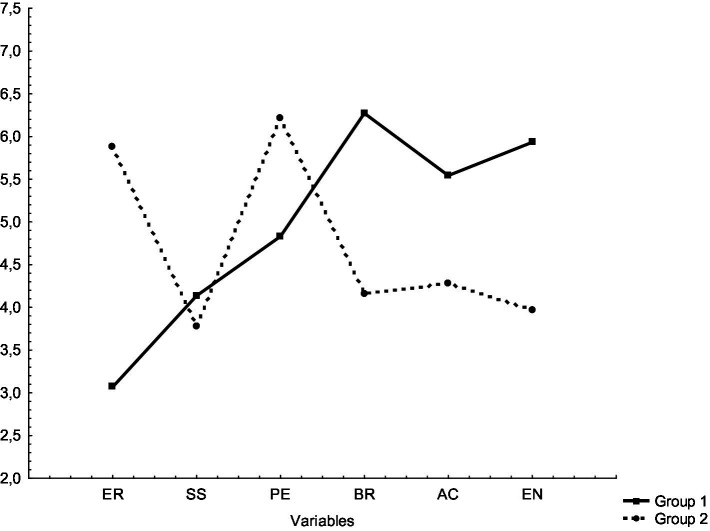
Temperament structure (trait profiles) of football players. ER, emotional reactivity; SS, sensory sensitivity; PE, perseverance; BR, briskness; AC, activity; EN, endurance.

Divided into two clusters, groups with different temperament trait profiles (temperament structure) were identified. The first group consisted of 59 individuals with trait levels close to the harmonized temperament structure with a high capacity to process stimulation, characterized by reduced levels of emotional reactivity, increased levels of briskness, activity and endurance. In the second group, there were 32 individuals with trait levels close to the harmonized temperament structure with a low capacity to process stimulation, characterized by increased levels of emotional reactivity and perseverance, decreased levels of briskness, activity and endurance (Figure 1).

Individuals in group 1 (higher capacity to process stimulation) had statistically significantly lower levels of emotional reactivity and perseverance, and higher levels of briskness, activity and endurance compared to individuals in group 2 (lower capacity to process stimulation). Only in terms of sensory sensitivity, there was no significant difference between the groups (Table 3).

**Table 3:** Comparison of the groups of football players separated in the cluster analysis in terms of the level of temperament traits.

Variables	Group 1 M (SD)	Group 2 M (SD)	*t*	df	*p*	Cohen d	Diff	95%CI	
								Low	Upp
ER	3.07 (1.28)	5.87 (1.48)	−9.44	89	<0.001	2.07	−2.81	−3.41	−2.20
SS	4.14 (1.73)	3.78 (1.77)	0.93	89	0.3569	–	0.35	−0.42	1.11
PE	4.83 (1.29)	6.22 (1.64)	−4.14	52.09	<0.001	0.98	−1.39	−2.05	−0.75
BR	6.27 (1.57)	4.16 (1.39)	6.36	89	<0.001	1.40	2.11	1.49	2.74
AC	5.54 (1.63)	4.28 (1.40)	3.69	89	<0.001	0.81	1.26	0.63	1.90
EN	5.93 (1.20)	3.97 (1.15)	7.56	89	<0.001	1.66	1.96	1.46	2.45

ER, emotional reactivity; SS, sensory sensitivity; PE, perseverance; BR, briskness; AC, activity; EN, endurance; M, medium; SD, standard deviation; *t*, Student’s *t*-test result; df, degrees of freedom; p, statistical significance level; Cohen d, indicator of effect size; Diff, difference; 95%CI, based on bootstrap percentile method using 10,000 samples; Low, lower bond; Upp, upper bond.

In addition, football players with higher capacity to process stimulation (Group 1) have statistically significantly higher levels of offensive, defensive (lower scores indicate higher effectiveness), and comprehensive efficiency compared to football players with lower capacity to process stimulation (Group 2) (Table 4.).

**Table 4:** Comparison of the specified groups of football players (temperament with higher and lower capacity to process stimulation) in terms of performance efficiency in 1x1 games.

Variables	Group 1 M (SD)	Group 2 M (SD)	*t*	df	p	Cohen d	Diff	95%CI	
								Low	Upp
Attack	10.10 (3.89)	6.50 (3.77)	4.26	89	<0.001	0.94	3.60	1.90	5.18
Deffence	7.88 (4.35)	10.56 (3.21)	−3.06	89	0.0029	0.67	−2.68	−4.21	−1.08
Effectiveness	2.22 (7.08)	−4.06 (5.60)	4.33	89	<0.001	0.95	6.28	3.56	8.83

Attack, attacking effectiveness; Deffence, defensive effectiveness; Effectiveness, comprehensive effectiveness; M, medium; SD, standard deviation; *t*, Student’s *t*-test result; df, degrees of freedom; p, statistical significance level; Cohen d, indicator of effect size; Diff, difference; 95%CI, based on bootstrap percentile method using 10,000 samples; Low, lower bond; Upp, upper bond.

## 4 Discussion

The aim of our study was to identify the temperament structures (functionally coupled traits distinguished by the RTT) that differentiate football players the most in terms of offensive, defensive, and comprehensive effectiveness during individual competition. The choice of a specific research goal is determined by the growing attention given to the study of players’ temperamental traits (Blecharz and Siekańska, 2007) and their importance in developing mental toughness (Wojciechowska-Maszkowska et al., 2020), managing stress and coping with excessive strain (Wirtz et al., 2007), as well as the effectiveness of attentional processes (Fajkowska et al., 2012).

Our own research has revealed that when analysing the players’ performance levels in individual competition (specifically in offensive, defensive and comprehensive indicator scores), they can be grouped into two distinct categories, representing different temperament structures. The first group, characterized by higher scores in offensive game (scoring more goals) and lower scores in defensive game (losing fewer goals), resulting in higher comprehensive effectiveness, exhibited lower levels of emotional reactivity and average perseverance, alongisde higher levels of briskness, activity, and endurance. This configuration of temperament traits can be described as harmonized structure with a high capacity to process stimulation, allowing effective regulation of stimulation and a general orientation toward stimulation seeking (Strelau and Zawadzki, 2014). Individuals with this structure demonstrate high adaptability, a lower tendency to dwell on past events, and greater resilience in stressful situations (Strelau, 2013; Strelau and Zawadzki, 2014). They are thus able to work for longer under the constant social evaluation that characterises the psychosocial demands placed on high-performance athletes, and to recover more quickly from an emotional setback or a mistake made during a game. In addition, individuals with this temperament structure do not require a structured action plan to feel comfortable (not reduce their own performance, and therefore that of the whole team) during activities, which can be crucial in the ever-changing conditions of a sports game.

Thus, it is indicated that those characterized by a harmonized structure with a high capacity to process stimulation could perform well in sports, such as for example: team sports games (requiring quick reactions and cooperation), tennis (quick decision-making), athletics (especially relay running) or synchronized swimming (requiring cooperation and coordination). At the same time, based on the description of people characterized by a certain temperament structure, it is indicated that in activities unrelated to sports, such people could be more likely to choose professional activities and tasks related to social interaction and the need to develop communicative competence (teacher, lawyer), aiming to solve creative tasks and requiring action for the integration of different social groups (event manager, trainers, animators).

On the other hand, the second group of players, scoring lower scores in the offensive game, higher scores in the defensive game, and consequently exhibiting lower comprehensive effectiveness, displayed higher levels of emotional reactivity and perseverance, and lower levels of briskness, activity, and endurance. This structure can be described as harmonized with a low capacity to process stimulation, characterising individuals who are inclined to discharge arousal and avoid stimulation. Consequently, they function better under conditions of low levels of stimuli (Strelau and Zawadzki, 2014). Such athletes tend to avoid highly stimulating activities in sporting situations, i.e., requiring high levels of effort or tolerating prolonged physical stimulation (prolonged games, actions aimed at quickly changing the outcome of a competition). Theoretically, they exhibit lower adaptability and cope less effectively with stress and in situations involving high variability of action (Strelau and Zawadzki, 2014). As a result, they may take longer to dwell on past failures or current setbacks, which can lead to a loss of concentration, motivation or increase the tendency to make more mistakes while competitions. In the context of intense one-on-one games, these factors contribute to a decrease in task effectiveness.

In sports individuals characterized by a harmonized with a low capacity to process stimulation could find themselves in such activities where high speed and pressure to act quickly is not required, such as for example: chess (requiring analytical thinking and patience), shooting (patience, precision), golf (concentration and patience) or long-distance running (ability to cope with loneliness during long runs, perseverance). In addition, in activities outside of sports, such people could successfully perform activities focused on analyzing and creatively interpreting data, understanding different contexts, or requiring a propensity for reflection and introspection (researchers, psychologists, writers, data analysts, programmers, architects). Highlighting the most significant differences between the two specified temperamental structures in our study, it is noteworthy that individuals with a harmonized temperamental structure, characterized by a high capacity to process stimulation (more effective in the individual game), demonstrate a greater adaptive potential compared to those with a low capacity to process stimulation (less effective in the individual game). The former primarily focuses on effectively regulating arousal and coping with constant social evaluation and perceived pressure. Conversely, the latter group tends to discharge arousal and avoid highly stimulating situations. These findings emphasise the importance of specific temperamental structures in realising potential and achieving optimal functioning. This has important implications for sports performance effectiveness since the effective regulation of arousal, conditioned by temperamental traits, plays a significant role in matching the level of incoming stimulation to an athlete’s ability to process it (Strelau and Zawadzki, 2014). In high-performance athletes, this balance should enable optimal performance without compromising cognitive control (Matczak and Knopp, 2013). It also helps athletes cope with the psychosocial demands of competition, allowing them to act in stressful situations where the source of stress can be either excessive or insufficient stimulation (Bołdak and Guszkowska, 2013). Moreover, individual reactions to stimulation can vary depending on personal, social, or geographical circumstances (Monasterio et al., 2016). Additionally, the temperamental structure is expected to support effective cooperating within one’s own team or when facing an opposing team (Paillard and Noe, 2006).

Referring to the most significant differences between the indicated temperament structures, it is determined that individuals who are described as more successful in the studied football activity (harmonized with a high capacity to process stimulation) are primarily characterized by a lower level of emotional reactivity and a higher level of briskness compared to less successful individuals (harmonized with a low capacity to process stimulation) in the one-on-one individual game. This suggests that effectiveness in the individual game, which involves stimulation, is largely influenced by reacting more efficiently to stimuli, adapting to environmental changes, maintaining a high pace of activity (corresponding to briskness as a temperament trait), and exhibiting a lower tendency to react intensely to emotionally evocative stimuli (corresponding to the concept of emotional reactivity) (Strelau and Zawadzki, 2014; Cyniak-Cieciura et al., 2018). These findings align with previous studies by: Bołdak and Guszkowska (2013), which observed predominately low and medium scores on the emotional reactivity scale in athletes, and Bojkowski and Siejka (2020), where emotional reactivity was identified as one of the temperamental traits differentiating competitive female football players based on their sports performance level (higher in low-performers). In light of these results, it is worth noting that low emotional reactivity is associated with composure, for instance, before important matches, and influences endurance (Łukaszewski and Marszał-Wiśniewska, 2006). It also helps individuals cope better with environment demands (Magier and Magier, 2015), more efficiently achieve goals without increasing arousal in critical circumstances, and employ task-based coping strategies for stress during dynamic and high-pressure activities. Additionally, significant relationships have been identified between emotional reactivity and reaction time, further confirming the role of these variables in the process of shaping psychomotor characteristics (Bernatowicz et al., 2015).

In summary, it is determined that the intensity of individual temperamental traits, organized in specific configurations (structures), enables athletes to adapt to the varying demands of stimulation inherent in their sport and subsequently influence their performance. In the context of football competition, this can also be linked to the effectiveness of the overall training process, which involves prolonged and stimulus-rich activities, as well as the initial competence characterized by pressure and strong stimuli. Considering that temperament structures determine the choice of human activities and their effectiveness, the appropriate selection of players for competition, taking into account their biologically determined characteristics, can serve as a foundation for facilitating the successful execution of individual, group, and team tasks.

The limitation of the self-study was the relatively small number of subjects studied and the sports level of the competing athletes (the group included participants in national competitions, but not national reps in international competitions). The result of the study may also have been influenced by the level of motivation of the athletes (not measured) to present the highest possible sports skills during the test game. For this reason, it is recommended that further research be conducted to determine the relationship of temperamental structures, including individual temperamental traits, with performance effectiveness (individual and team) in soccer players characterized by the highest possible sporting level (international), as well as those competing in different tactical positions (defenders, midfielders and strikers). Among the prospects for future research, one can also point to the relevance of conducting longitudinal studies to indicate the relationship of temperament structures to sports development and cross-cultural within temperament structures and their relationship to sports performance.

The practical aspect of the research carried out may concern the identification of players’ potential and selection for team sports games, the individualization of sports training (adaptation of training programmes or mental preparation plans), including attention to the needs of individual players, the interpersonal communication aspects used, the processes of analysis of mistakes made or the resources possessed. These aspects can help both in supporting the personal development of players and in the process of building effective teams (interaction between players).

## Data availability statement

The raw data supporting the conclusions of this article will be made available by the authors, without undue reservation.

## Ethics statement

The studies involving humans were approved by the Research Ethics Committee of the Karol Marcinkowski University of Medical Science in Poznań (approval number 780/14; date: 02.10.2014). The studies were conducted in accordance with the local legislation and institutional requirements. Written informed consent for participation in this study was provided by the participants’ legal guardians/next of kin.

## Author contributions

ŁB: Conceptualization, Data curation, Formal analysis, Funding acquisition, Investigation, Methodology, Project administration, Resources, Software, Visualization, Writing – original draft, Writing – review & editing. MT: Conceptualization, Formal analysis, Investigation, Methodology, Project administration, Software, Supervision, Validation, Visualization, Writing – original draft, Writing – review & editing.
